# Study of thyroid function among COVID-19-affected and non-affected people during pre and post-vaccination

**DOI:** 10.1186/s12902-022-01187-0

**Published:** 2022-12-09

**Authors:** Mamudul Hasan Razu, Md. Iqbal Hossain, Zabed Bin Ahmed, Mousumi Bhowmik, Md. Kazy Ebnul Hasan, Md. Kaderi Kibria, Dil Afroj Moni, Mala Khan

**Affiliations:** Bangladesh Reference Institute for Chemical Measurements (BRiCM), Dhaka, Bangladesh

**Keywords:** COVID‑19, SARS-CoV-2 vaccine, TSH, TT3, RT-PCR

## Abstract

The novel coronavirus COVID-19 has caused a global pandemic with many long-ranging effects on the physiological balance of the human body. The impact of COVID-19 on the thyroid axis remains uncertain. Our aim was to assess the long-term consequences of COVID-19 infection and its vaccination with thyroid hormones. Thirty laboratory-confirmed COVID-19-positive patients with no vaccination record, thirty COVID-19-negative patients with vaccination records, and ten healthy subjects were retrospectively, and cross-sectionally enrolled in this study. An ELISA assay was performed to evaluate thyroid function tests, including the total triiodothyronine (TT3), total thyroxine (TT4), and thyroid stimulating hormone (TSH). We found decreased levels of TT3, average or low plasma T4 levels, and standard or slightly decreased TSH levels in unvaccinated COVID-19-positive patients than in the healthy group, while the vaccinated COVID-19-negative group had normal thyroid hormone levels compared to controls. The correlation between TT3 and TSH levels gradually shifted from no association to a negative pattern in the unvaccinated COVID-19-positive group. Again, a highly significant negative correlation between TSH and TT3 was observed on days above 150, although a slight fluctuation was noted on day 90. This pilot study from Bangladesh shows that abnormalities in thyroid function can be observed during COVID-19 infection and after vaccination, which gradually recovers over time.

## Introduction

Since the emergence of the COVID-19 pandemic, an exponentially growing dataset shows that SARS-CoV-2 infection affects multiple organs with short- and long-term consequences [1]. The available studies suggest that COVID-19 can affect the endocrine organs with numerous clinical manifestations due to cytokine and chemokine secretion, vascular disorders, and autoimmune reactions [2, 3]. Pathologies related to the pancreas, pituitary, gonads, and thyroid can occur by different mechanisms [4].

**Fig. 1:**
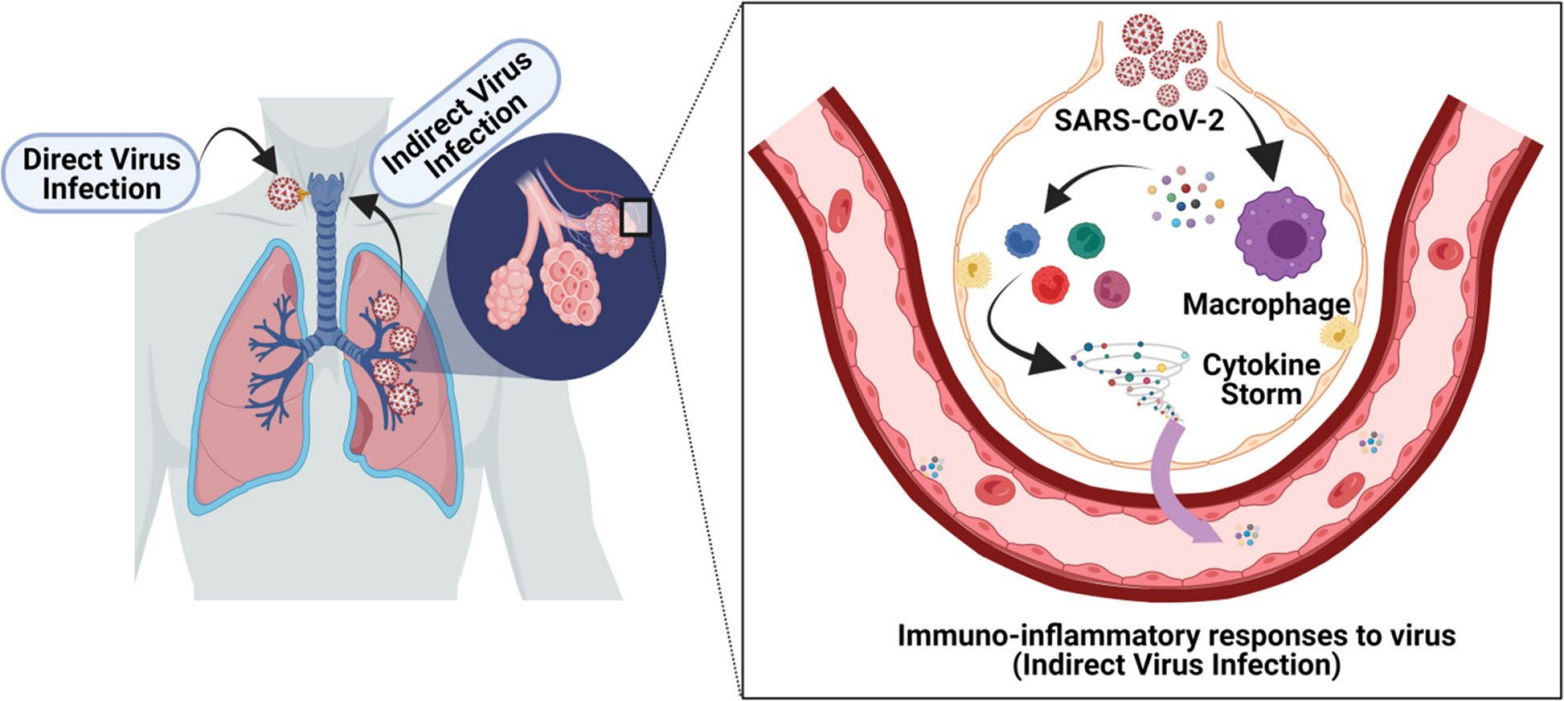
Schematic representation of the possible mechanisms of COVID-19 infection in thyroid gland

It is known that the thyroid gland and viral infection are involved in a complex interaction via hormones and immunomodulatory signaling molecules. These relations have been established in physiological and pathological contexts (Fig. 1) [5, 6]. Viruses with associated inflammatory immune responses could be considered an important variable affecting lifelong thyroid function and consequently contribute to the definition of thyroid biography at the individual level [7]. The impact of viruses on thyroid function can undoubtedly lead to multisystem damage, as thyroid hormone affects the development and function of virtually all human cells, including neural maturation of olfactory receptor neurons [8]. There is currently conflicting evidence about the effect of COVID-19 on thyroid function. A prospective observational study by Lui et al. found abnormal thyroid function tests, defined as impaired thyroid-stimulating hormone (TSH) and/or free thyroxine (fT4) and/or free triiodothyronine (fT3), in 25 patients (13.1%), suggesting SARS-CoV-2 could directly induce viral thyroiditis. In addition, low fT3 levels were independently associated with an increased likelihood of clinical deterioration. The researchers concluded that there may be a direct effect of SARS-CoV-2 on thyroid function, possibly leading to an exacerbation of pre-existing autoimmune thyroid disease [9].

On the other hand, Chen et al. [10] conducted a study of 50 patients with COVID-19 in China. They demonstrated an overall decrease in TSH, total thyroxine (T4), and triiodothyronine (T3), more consistent with a nonthyroidal disease pattern. Moreover, some cases have also reported other COVID-19-related thyroid disorders, such as thyrotoxicosis and hypothyroidism [11].

Several types of vaccines were approved during the catastrophic COVID-19 pandemic, and the early literature on vaccination campaigns documented a satisfactorily high protection and safety profile against the disease [12]. However, as the vaccination program progressed, some cases were reported suggesting a possible association between thyroid disease (e.g., subacute thyroiditis, Graves' disease, focal painful thyroiditis, silent thyroiditis, thyroid-ocular disease, etc.) and vaccination [13]. Although these studies provide useful information, the effects of COVID-19 infection and vaccination on the thyroid axis are contradictory.

We conducted a retrospective, cross-sectional study of COVID-19-positive and COVID-19-negative subjects compared to a matched healthy control group. We aimed to determine whether the COVID-19 infection and vaccination cause thyroid abnormalities as determined by a diagnostic assessment of thyroid function.

## Materials and methods

This retrospective and cross-sectional study was conducted at the Bangladesh Reference Institute for Chemical Measurements (BRiCM), Dhaka, Bangladesh, and approved by the BRiCM's Institutional Research Ethics Committee (reference number: BRiCM 2206). The research was conducted ethically according to the World Medical Association of Helsinki Declaration. Written informed consent was obtained from all participants. The medical records of 30 patients with laboratory-confirmed COVID-19 were retrospectively examined, and they had not received the COVID-19 vaccine. A “confirmed case” was defined as “an individual with laboratory confirmation of COVID-19 infection by RT-PCR testing, regardless of clinical signs and symptoms.” Healthy participants with no thyroid disease or other medical history that might impair thyroid function were included as a control group. To determine if COVID-19 vaccination has unique effects on thyroid function, we included another group of 30 vaccinated COVID-19-negative patients with similar severity. Vaccination cards were collected from COVID-19-negative patients during the sampling period. Participants with no COVID-19 infection and vaccination history were included as healthy controls, and who were unvaccinated COVID-19 positive and vaccinated COVID-19 negative were included as cases. Exclusion Criteria: Patients with a history of thyroid disease, without assessment and follow-up of thyroid function, autoimmune disease, and pregnancy were excluded from the study. Participants who could not provide an RT-PCR report and vaccination status were also excluded from the study. The blood samples were collected at BRiCM collection booth over time followed by the determination and quantification of thyroid hormones. Triiodothyronine (TT3) (reference range: 70 to 200 ng/dL), thyroxine (TT4) (reference range: 4.6–11.2 mcg/dL), and thyroid stimulating hormone (TSH) (reference range: 0.45–4.5 mIU/L) were measured in all groups successfully. Multiskan™ FC Microplate Photometer was used for the ELISA assay to determine hormone levels in human serum. All parameters were analyzed by QikTech ELISA Kit (San Diego, CA 92121, U.S.A) and compared between groups. Any deviation from the normal reference range was considered thyroid dysfunction. We also collected the clinicopathological characteristics of participants' age, gender, and medical history, and compared them in unvaccinated COVID-19 positive, vaccinated COVID-19 negative, and healthy subjects. We observed the critical changes in thyroid function over time. We categorized study participants into Group A (healthy participants), Group B (unvaccinated COVID-19 positive participants), and Group C (vaccinated COVID-19 negative participants).

Statistical analysis was performed using SPSS (version 26) (SPSS Inc., Chicago, IL, USA) and R Language (version 4.2.0). Quantitative data were presented as mean ± standard deviation (SD), and qualitative data as frequencies and percentages. The students’t-test and analysis of variance (ANOVA) were used to analyze all subjects' characteristics and thyroid function. The correlation between TSH, TT3, and TT4 was compared using the Wilcoxon rank-sum test and Pearson's correlation. For all analyses, *p* < 0.05 was considered statistically significant.

## Results

A total of 70 respondents were enrolled in our retrospective and cross-sectional study. These included an equal number of patients with unvaccinated confirmed COVID-19 positive and vaccinated COVID-19 negative status. Ten healthy subjects were also included as controls. The demographic characteristics of the respondents are presented in Table 1. The average age and weight of the respondents were 35.30** ± **15.60 years and 63.54 ± 12.36 kg. Almost all professions were recruited in this study, and the disease status of the respondents was observed. 18.60% of the total respondents had high blood pressure, followed by diabetes (13.95%) and a previous history of dengue (13.95%), asthma (11.62%), and allergies (11.62%), fever (6.97%) and cardiovascular disease (6.97%), and 2.32% of them had other problems. The respondents' average TSH, TT3, and TT4 were 3.96 ± 0.27, 0.70 ± 0.05, and 4.78 ± 0.15, respectively. We found mild to severe COVID-19 infection in the unvaccinated COVID-19-positive group.

**Table 1:** Demographic characteristics of the respondents (*n* = 70)

Characteristics	Mean ± SD or n(%)	Characteristics	Mean ± SD or n(%)
Age (years)	35.30** ± **15.60	Gender	
Weight (kg)	63.54 ± 12.36	Male	38 (55.1%)
Occupation		Female	31 (44.9%)
Architect	1 (1.4%)	Different Diseases	
Banking	2 (2.8%)	Asthma	5 (11.62%)
Business	10 (14.5%)	Diabetes	6 (13.95%)
Cricketer	1 (1.4%)	HBP	8 (18.60%)
Designer	1 (1.4%)	LBP	2 (4.65%)
Physicians	4 (5.8%)	Allergy	5 (11.62%)
Driver	1 (1.4%)	Fever	3 (6.97%)
Ex Banker	1 (1.4%)	Cold	1 (2.32%)
Housewife	10 (14.4%)	CAD	3 (6.97%)
Private	5 (7.2%)	Nephrological	1 (2.32%)
Service	9 (13%)	Liver	1 (2.32%)
Student	7 (10.1%)	Cancer	1 (2.32%)
Teacher	7 (10.1%)	PDH	6 (13.95%)
Psychiatric	1 (1.4%)	Anemia	1 (2.32%)
Daily Labour	9 (13%)		
Thyroid Function Information		COVID-19 Severity	
TSH	3.96** ± **0.27	Mild	10 (55.55%)
TT3	0.70** ± **0.05	Moderate	7 (38.88%)
TT4	4.78** ± **0.15	Severe	1 (5.55%)

*HBP* High blood pressure, *LBP* Low blood pressure, *CAD* Cardiovascular diseases, *PDH* Previous dengue history

**Fig. 2:**
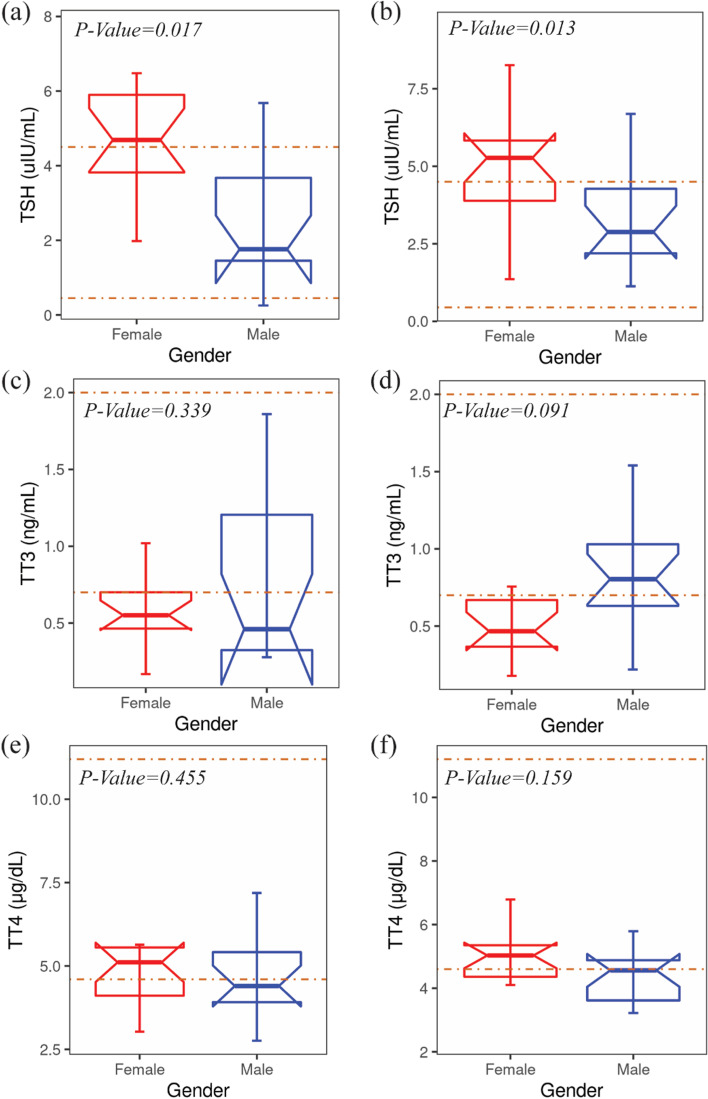
Gender-wise comparison of the TSH, TT3, and TT4 levels between Group B and Group C. (a,b) TSH levels, (c,d) TT3 levels, and (e,f) TT4 levels between female and male of both groups

The gender-wise comparison of thyroid function was observed, and it showed that the TSH level was significantly high in both Group B and Group C female patients (*p*-value = 0.017 and 0.013, respectively) compared to male groups with both conditions. Moreover, the TSH level in female patients of both groups exceeded the upper standard limit of thyroid stimulating hormone Fig. 2a and b. TT3 (Fig. 2c and d) and TT4 (Fig. 2e and f) between groups did not show any significant difference.

Further, we compared the TSH, TT3, and TT4 levels among Group A, B, and C (Table 2). We observed that TSH levels were significantly higher (*p*-value < 0.05) in both Group B and C than in the control group A, while the TT3 and TT4 levels were substantially lower (*p*-value < 0.05) in both groups compared to the healthy controls.

**Table 2:** Comparison of thyroid function among Group A, B, and C patients

Thyroids function	Group A (*n* = 10)	Group B (*n* = 30)	Group C (*n* = 30)	*p*-value
TSH	2.411 ± 0.352	3.96 ± 0.37	4.17 ± 0.39	0.003
TT3	1.41 ± 0.064	0.67 ± 0.07	0.72 ± 0.07	0.000
TT4	6.88 ± 0.241	4.86 ± 0.24	4.65 ± 0.18	0.000

Significantly different between groups A, B, and C at *P* < 0.05

**Fig. 3:**
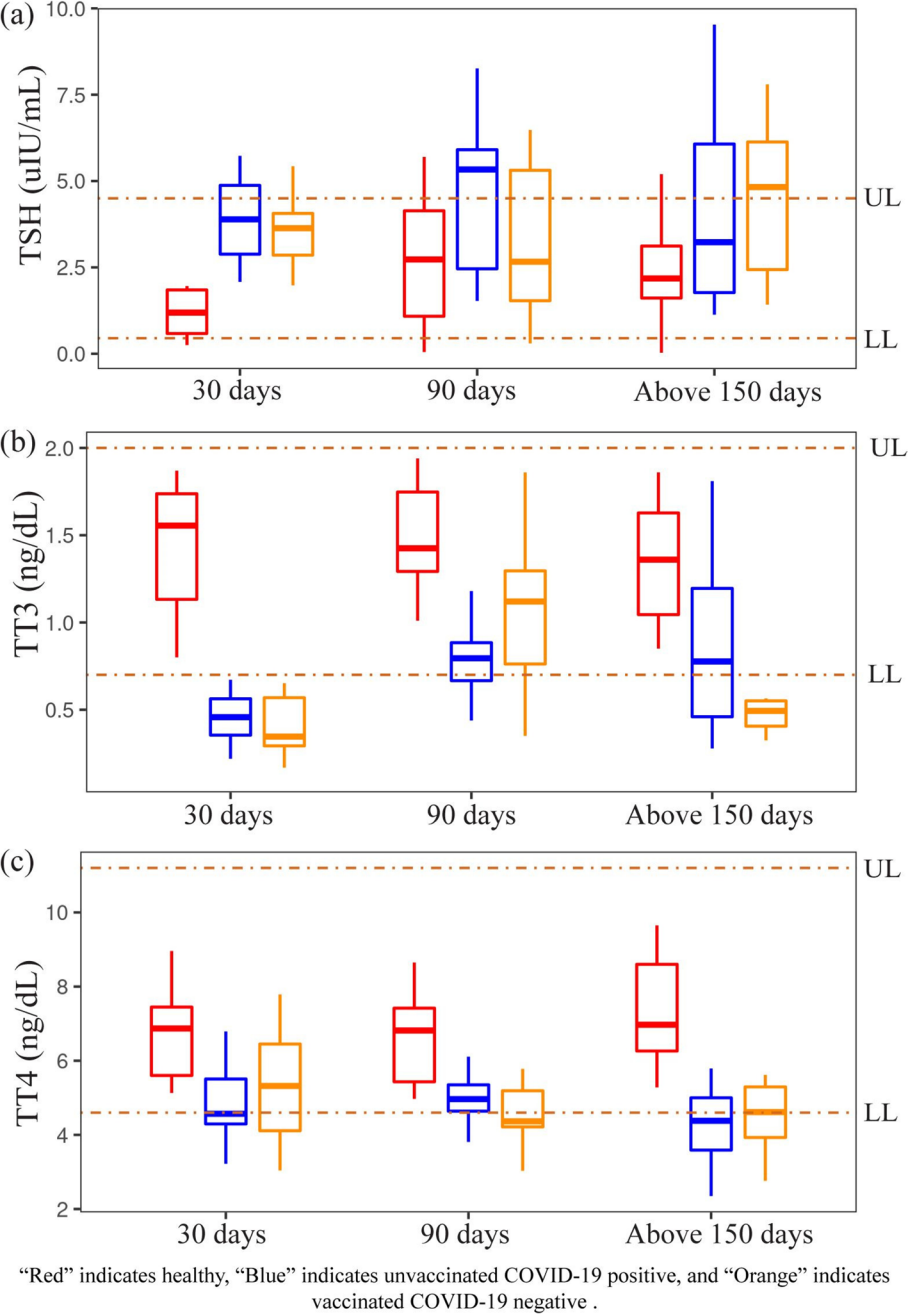
Thyroid functions of Group A, B and C in the time intervals of 1, 3 and above 5 months

Next, we constructed a boxplot (Fig. 3) to demonstrate the thyroid functions of the Group A, B, and C at one, three, and more than five-month intervals. In the healthy controls (Group A), TSH, TT3, and TT4 levels were within the normal range with minor differences from the first month to more than five months. In the case of the Group B, (a) the TSH level increased after third month compared to the first month, and then it started decreasing at above five months, and the difference was not statistically significant (*p* > 0.05), but the median TSH level exceeded the upper limit of normal range after third months. (b) The TT3 level increased during the third and five months compared to the first month, and the difference was not statistically significant (*p* > 0.05). Although the median TT3 level exceeded the lower limit of the normal range in the first month, it was within the normal range after the third and fifth months. (c) TT4 level also increased in the third month compared to the first month but decreased after five-month than in the first and the third month, and the difference was not statistically significant (*p* > 0.05). Still, after five months, the median TT4 level was lower than the normal range. Again, in Group C, (d) the TSH level decreased in the third month than in the first, but after more than five months, it increased than in the first. Though their differences were not statistically significant (*p* > 0.05), the median TSH value also exceeded the normal range after five months. (e) TT3 level was remarkably higher in the third month than in the first; after five months, it decreased, and their differences were statistically significant (*p* < 0.05). On the other hand, the median TT3 exceeded the normal range for all except the third month. (f) TT4 levels were relatively low after the third and more than five months than in the first month, and the difference was insignificant (*p* > 0.05).

**Fig. 4:**
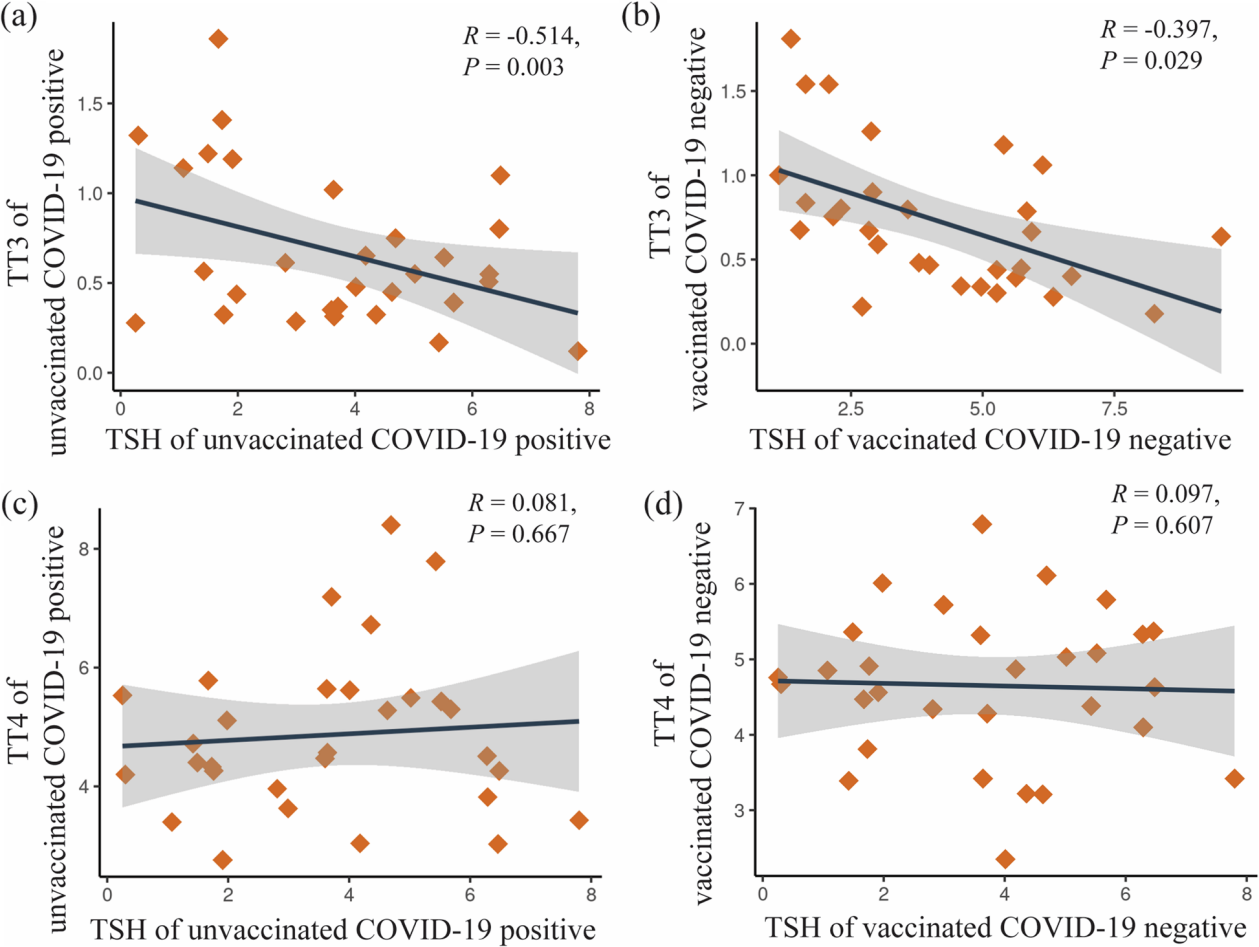
The relationship of TSH with TT3 and TT4 in Group B and C

After elucidating thyroid functions between groups, correlation plots were constructed to illustrate the relationship of TSH to TT3 and TT4 in Group B and Group C. The correlation analysis revealed that TSH and TT3 levels in both Group are inversely correlated with a significant p-value (*p* = 0.05) (Fig. 4a and b). On the other hand, the correlation coefficients between TSH and TT4 levels in the two groups do not correlate with each other at a high *p*-value (Fig. 4c and d).

**Fig. 5:**
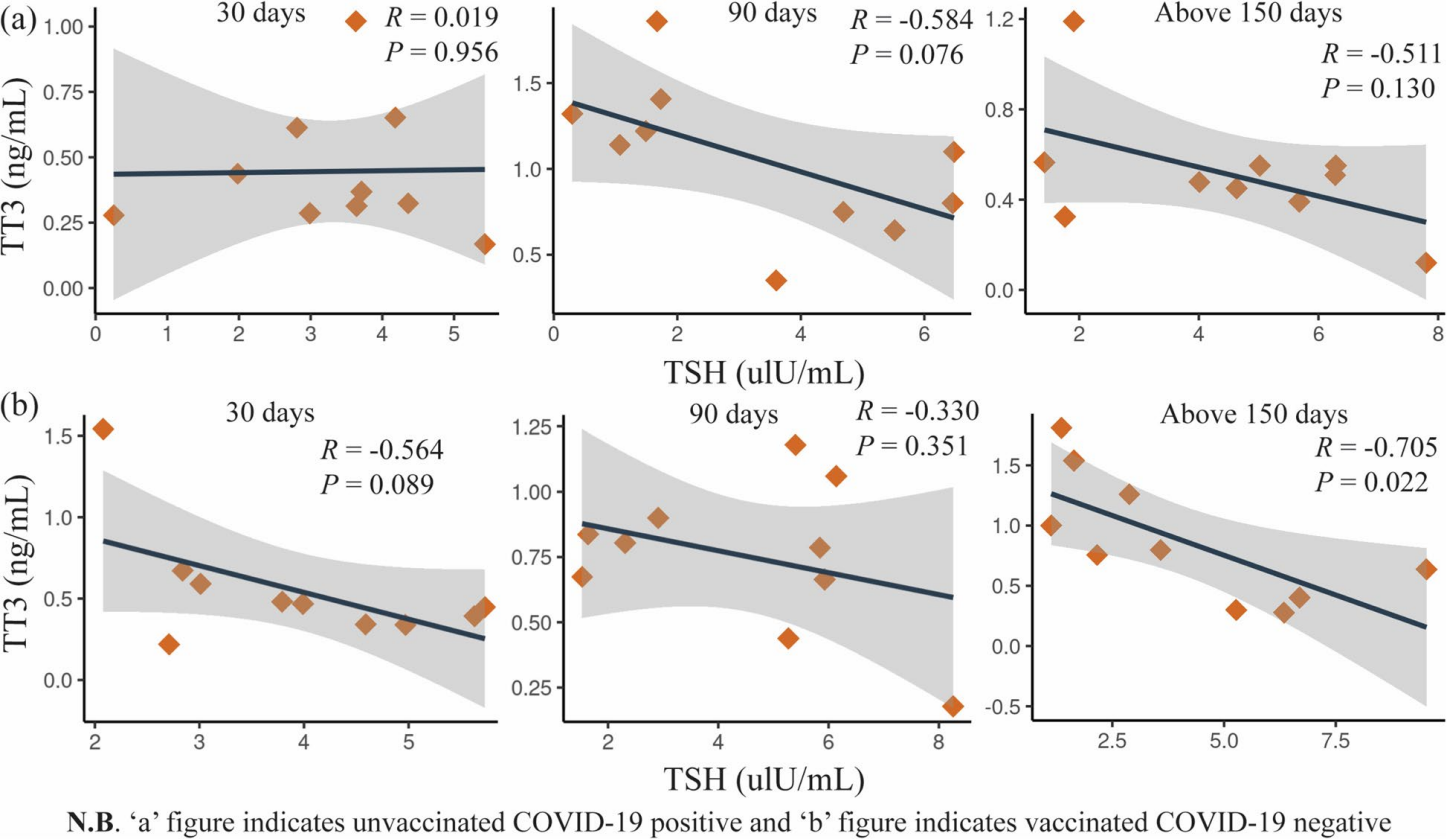
Month wise association between TSH and TT3 level in Group B and C patients

We then focused our research on TSH and TT3. We investigated the month-wise correlation of TSH and TT3 levels at different time duration in patients of Group B and C. Figure 5a depicts the association between TSH and TT3 levels in Group B patients at 1-month, 3- month, and above 5-month intervals. TSH and TT3 levels tend to have no association at day 30 and turn toward reciprocally correlating at day 90 (R = -0.584) and day above 150 (R = -0.511), but the p-value is not statistically significant at the 5% level. Similarly, the association between TSH and TT3 levels in Group C in a different timeframe (Fig. 5b) showed that the association tended to be inversely correlated at day 30 (R = -0.564). Although the association turned towards a weak negative state at day 90, a high negative correlation between them was observed at days above 150 with a significant *p*-value (*p* < 0.05).

## Discussion

COVID-19 is an infectious disease that has caused a global pandemic. As a novel type of disease with a high risk of infection and mortality, the pathophysiology of COVID-19 is not yet fully understood. A number of studies have reported severe and complex effects of COVID-19 on multiple human organs and systems, including immune, respiratory, circulatory, digestive, hepatic, renal, and hematological systems [14]. However, the influence of the COVID-19 virus on thyroid hormone levels and the underlying mechanisms are still unclear. Here, we investigated thyroid hormone levels among unvaccinated COVID-19-positive and vaccinated COVID-19-negative people during pre-and post-vaccination. Our study samples randomly selected healthy controls (Group A), unvaccinated COVID-19 positive (Group B), and vaccinated COVID-19 negative (Group C) patients. We found that patients from group B presented significantly higher levels of TSH and lower levels of TT4 than healthy subjects. However, the levels among groups were within the normal range. We also observed that the TT3 level was significantly lower in the group B patients than in the control group. These criteria mimic the pattern observed in patients with non-thyroidal disease (NTI).

Non-thyroidal disease syndrome presents as abnormal thyroid function in serious diseases other than thyroid disease, including severe infections, cardiovascular and gastrointestinal disorders, severe diabetic complications, malignant tumors, severe malnutrition, burns, and trauma [15]. It is well established that NTI is a consequence of an acute phase response to macronutrient restriction or severe systemic disease, and the most typical alterations are decreased plasma triiodothyronine (T3) level, normal or low plasma thyroxine (T4) level, and standard or slightly decreased TSH level [15, 16]. The decreased T3 and normal TSH phenomenon in COVID-19 patients was consistent with NTI. On the other hand, some cases of post-vaccination thyroid complications have been observed after the administration of different types of COVID-19 vaccines [4, 17–20]. Some studies reported subacute thyroiditis following COVID-19 vaccination [4, 20]. Similarly, Vera-Lastra et al. reported two cases of Grave’s disease three days after SARS-CoV-2 vaccination [18], and they mentioned that adjuvants might induce the disorder. However, in our study, Group C showed normal thyroid hormone levels, in contrast to the abovementioned studies. One of the possible explanations may be that most of the complications were observed immediately after vaccination. Since our study samples were taken with a time intervals, hormone levels could return to normal.

We continued our further analysis of thyroid functions between group B and C at 1, 3, and more than 5 months. Interestingly, the TSH, TT3, and TT4 levels showed fluxes ranging from low to high and vice-versa from day 30 to day above 150 compared to the healthy control, and levels were almost within the borderline ranges after days 150 or above. Wang et al. conducted their study on seven patients with lower than-normal TSH and TT3 levels upon admission, which became normalized by day 30. In addition, the malfunction feedback between TT3 and TSH returned to work overtime [21]. A recent case report of thyroiditis after SARS-CoV-2 infection came from Brancatella et al. confirmed this hypothesis. That case presented thyroid dysfunction followed by a triphasic course, including hypothyroidism, thyrotoxicosis, and euthyroidism, and then recovered to normal within a month [22]. Two other studies from China and Italy found lower TSH levels in more severely affected patients with COVID-19 and thyrotoxicosis, respectively, after a confirmatory diagnosis of COVID-19, but all thyroid function tests returned close to baseline at follow-up in both Chinese and Italian studies [10, 23]. Changes in iodothyronine deiodinase levels, TSH secretion, binding of thyroid hormone to plasma proteins, transport of thyroid hormone to peripheral tissues, and changes in thyroid hormone receptor activity are thought to all likely contribute to the changes in serum levels of thyroid hormone in COVID -19 patients. Still, this needs further investigation [24].

Moreover, the correlation analysis by Wang et al. initially observed a positive pattern that changed to a negative one over time, although the p-values ​​were statistically insignificant [21]. In our present study, we also analyzed the correlation of TSH with TT3 in Group B and C. We found that the TSH and TT3 levels shifted from no association to a negative pattern in Group B. However, the p-values did not reach statistical significance, given the limited number of patients. On the other hand, in Group C, a highly significant negative correlation was observed between TSH and TT3 at days above 150. However, a minor fluctuation of weak correlation was detected at day 90. These features indicated a recovery of the pituitary-thyroid axis.

This study had some limitations. One was a small number of sample sizes, and random sample collection could introduce potential bias. Second, participants in the vaccinated COVID-19 negative group were reported taking Covishield (AstraZeneca). A variation in vaccine types could help us more accurately assess post-vaccination efficacy. For a precise result, a good number of samples and long-term follow-up is recommended.

## Conclusion

In conclusion, the current study demonstrated that thyroid functions can be disrupted between COVID-19 infection and vaccination phases. Thyroid hormone levels appear to change dynamically and recover gradually and spontaneously. Although some evidence suggests that thyroid dysfunction can be caused after COVID-19 infection and vaccination, the pathogenesis has not been unequivocally characterized. However, more research is needed at the molecular and clinical levels to better understand the effects of SARS-CoV-2 infection and vaccination with thyroid hormone function. In parallel, physicians must practice clinical awareness when monitoring for recovery from COVID-19 disease and vaccination effects for signs and symptoms of thyroid hormone imbalances.

## Data Availability

The datasets used and/or analyzed during the current study are available from the corresponding author on reasonable request- bricmdg@yahoo.com.
